# Study on administration of 1,5-anhydro-D-fructose in C57BL/6J mice challenged with high-fat diet

**DOI:** 10.1186/1472-6823-10-17

**Published:** 2010-10-19

**Authors:** Jie Mei, Shukun Yu, Bo Ahrén

**Affiliations:** 1 Department of Medicine, B11 BMC, S-221 84, Lund University, Lund, Sweden; 2 Department of Biotechnology, Box 124, S-221 00, Lund University, Lund, Sweden; 3 Enzyme R&D, Genencor Division, Danisco A/S, Edwin Rahrs Vej, 38, Brabrand, DK 8220, Denmark

## Abstract

1,5-Anhydro-D-fructose (AF) is a mono-saccharide directly formed from starch and glycogen by the action of α-1,4-glucan lyase (EC 4.2.2.13). Our previous study has indicated that AF increases glucose tolerance and insulin secretion in NMRI mice after administration through a gastric gavage in a single dose at 150 mg per mouse. In this study, we used high-fat feeding of C57BL/6J mice to examine the influence of long-term administration of AF on glucose-stimulated insulin secretion *in vivo *and *in vitro*. We found that 8-weeks of high-fat feeding increased body weight, fasting blood glucose and insulin levels in C57BL/6J mice when compared to mice fed normal diet. Impaired glucose tolerance was also observed in mice receiving 8-weeks of high-fat diet. In contrast, AF (1.5 g/kg/day), administered through drinking water for 8-weeks, did not affect body weight or food and water intake in mice fed either the high-fat or normal diet. There was no difference in basal blood glucose or insulin levels between AF-treated and control group. Oral glucose tolerance test (OGTT) showed that AF did not affect glucose-stimulated insulin secretion in mice. In *in vitro *studies with isolated islets, AF did not influence glucose-stimulated insulin secretion in mice receiving either high-fat or normal diet. We therefore conclude that when given through drinking water for 8 weeks at 1.5 g/kg/day, AF has no effect on glucose-stimulated insulin secretion in C57BL/6J mice challenged with a high-fat diet.

## Background

1,5-Anhydro-D-fructose (AF) is a mono-saccharide having structural similarity to glucose [1]. It is produced by the degradation of starch and glycogen catalysed by the enzyme α-1,4-glucan lyase [1]. AF is present in fungi and algae, including edible fungal and algal species [2,3], as well as in mammalian tissues including rat liver [1,4]. *In vitro *studies have indicated that enzymatic oxidation of 1,5-anhydro-D-glucitol (AG) by fungal pyranose 2-oxidases results in the formation of AF; however, this reaction has not been demonstrated *in vivo *in mammals [5]. In mammals, the further metabolism of AF involves a NADPH-dependent specific reductase that reduces AF to AG [1,6]. It has been reported that AG, the second most abundant polyol after glucose in human fluid, stimulated insulin secretion in two rodent insulinoma cell lines studied, *i.e.*, rat RINr and mouse MIN6 at physiological relevant concentrations [7]. In fungi and red algae AF is metabolised to secondary metabolites such as microthecin, ascopyrones and echinosporin [2,3,8,9]. However, the importance of AF in mammalian physiology remains elusive. The works by Hisaku *et al*. [10], Fujisie *et al*. [11] and Yamaji *et al. *[12] have indicated that AF has antioxidant and antimicrobial effects, suggesting a potential biological role for AF in mammals. Furthermore, we have previously shown that when given through a gastric gavage (150 mg) together with glucose (150 mg/mouse), AF induces glucose tolerance, insulin secretion and increases in plasma levels of glucagon-like peptide-1 (GLP-1) [13]. The effect of AF on glucose tolerance, however, was not detected when administered intravenously [13]. Based on these observations, the role of AF in increasing endogenous GLP-1 secretion needs to be explored further to clarify the discrepancy. In the current study, we used high-fat feeding of C57BL/6J mice as a model to investigate the effect of long-term administration of AF on glucose-stimulated insulin secretion *in vivo *and *in vitro*. C57BL/6J mice are susceptible to high-fat diet and develop glucose intolerance more readily than other strains [14]. Furthermore as indicated above, as AF metabolism is an energy-consuming process due to the use of NADPH in its reduction to AG [1,6], feeding mice with AF might reduce the extent for obesity development.

## Methods

### Animal

Four-week old female C57BL/6J mice weighing 15 g were obtained from Bomholtgaard Breeding and Research Center, Denmark. Animals were housed on a 12-h light/dark cycle with *ad libitum *access to diets and water. The mice were fed with either a standard rodent food or a high-fat diet (#D12310 and #D12309; Research Diets, New Brunswick, NJ). The normal diet had a caloric density of 12.6 kJ/g and contained 25.8% protein, 62.8% carbohydrates and 11.4% fat. The high-fat diet consisted of 16.4% protein, 25.6% carbohydrates and 58.0% fat with a caloric density of 23.6 kJ/g. The mice remained on each of the diets for 8 weeks. During the 8 weeks, AF (1.5 g/kg/day) dissolved in tap water was made accessible to the mice. The control group received tap water. The food and water intake and body weight were recorded weekly.

### Oral glucose tolerance test

After 8 weeks of AF treatment, blood was drawn from the intra-orbital bullar plexus of all mice for the measurement of basal glucose and insulin levels. For oral glucose tolerance test (OGTT), mice fasting overnight were given glucose (150 mg/mouse) orally and their blood was collected at times 0, 15, 30, 60, 90 and 120 min following glucose administration. All procedures using animals were approved by the local Ethics Committee and followed the guidelines for experimentation in animals (European Economic Community Council Directive 86/609/EEC).

### Insulin secretion *in vitro*

Pancreatic islets were isolated from mice using the collagenase isolation technique. Briefly, the common bile duct was ligated at the papilla vateri and cannulated after a midline incision. The pancreas was filled with 3 ml of ice-cold Hank's balanced salts (HBSS) supplemented with 0.4 mg/ml collagenase P (Roche Molecular Biochemicals, Mannheim, Germany) before removal and then incubated at 37°C for 19 min. After washing the incubated islets for three times with HBSS, they were handpicked under a stereomicroscope and incubated overnight in RPMI 1640 medium supplemented with 10% fetal bovine serum, 2.05 mmol/l L-glutamine, 2.5 μg/ml amphotericin B, 100 IU/ml penicillin and 100 μg/ml streptomycin at 37°C in humidified air equilibrated with 5% CO_2_. After overnight incubation, the islets were washed three times and then pre-incubated for 60 min at 37°C in a Hepes medium (pH 7.4 supplemented with 0.1% human serum albumin (Sigma) and 3.3 mmol/l glucose. The Hepes medium consisted of 125 mmol/l NaCl, 5.9 mmol/l KCl, 1.2 mmol/l MgCl_2_, 1.28 mmol/l CaCl_2 _and 25 mmol/l Hepes. After pre-incubation, groups of three islets were transferred into separate chambers containing 200 μl of the Hepes or RPMI medium supplemented with glucose at various concentrations. Following incubation at 37°C for 60 min, 25 μl of the medium was collected and stored at -20°C until analysis.

### Analysis of insulin and glucose

Insulin was determined using guinea pig anti-rat insulin, ^125^I-labeled human insulin, and rat insulin standard (Linco Research Inc, St Charles, MO) by RadioImmunoAssay (RIA). Plasma glucose was determined by the glucose oxidase method.

### Statistics

Mean values ± SE for glucose and insulin plasma levels are shown. Statistical comparisons for differences between AF and control were performed by unpaired Student *t *test.

## Results

### AF treatment for 8 weeks

As seen in Table 1, 8 weeks of high-fat feeding increased body weight and blood glucose compared with the mice fed normal diet. By contrast, AF did not change/reduce body weight, blood glucose and insulin levels in mice fed high-fat diet. There was no difference in the food and water intake levels between AF-treated and control group of mice fed high-fat diet. In mice fed normal diet, AF did not affect body weight, food and water intake, or fasting blood levels for glucose and insulin (Table 1).

**Table 1:** Body weight, food intake and water intake in C57BL/6J mice treated with AF for 8 weeks.

High-fat diet	Normal diet	
(8 weeks)	(8 weeks)	
**Body weight (g)**		
Control	28 ± 1.0	21.2 ± 0.1*
AF	30.4 ±1.3	21 ± 0.2*
**Food intake (g)**		
Control	2.1 ± 0.05	2.5 ± 0.07
AF	2.2 ± 0.29	2.6 ± 0.04
**Water Intake (ml)**		
Control	3.4 ± 0.8 3.1 ± 0.9	
AF	3.8 ± 0.9	3.5 ± 1.0
**Glucose (mmol/l)**		
Control	7.4 ± 0.3	5.4 ± 0.4*
AF	7.8 ± 0.4	5.3 ± 0.3*
**Insulin (pmol/l)**		
Control	211 ± 22	129 ± 26*
AF	302 ± 34	113 ± 30*

Data are means ± SE (n = 12). Student's *t *test, *p < 0.05 between high-fat diet and normal diet.

### Oral glucose tolerance test (OGTT)

**Figure 1:**
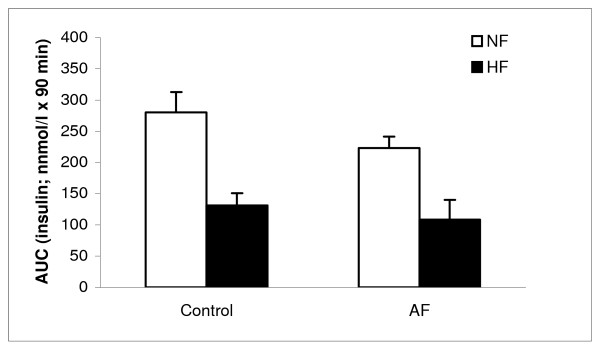
**Islet hormone secretion *in vivo***. The AUC _insulin _for 90 min is calculated after oral glucose tolerance test. HF, in high-fat diet, NF, in normal diet. Data are means ± SE (n = 12).

**Figure 2:**
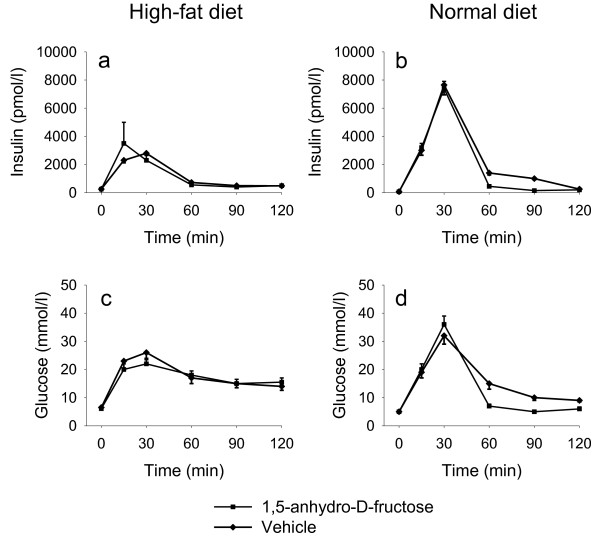
**Oral glucose tolerance test (OGTT) in C57BL/6J mice treated with AF for 8 weeks in high-fat diet and in normal diet**. Overnight-fasted mice were administrated 1.0 g/kg glucose by a gastric gavage. Blood samples were taken at 0, 15, 30, 60, 90 and 120 min. Plasma levels of insulin (a, b) and glucose (c, d) were measured. Data are means ± SE (n = 12).

After 8 weeks of high-fat or normal diet, oral glucose tolerance test was performed in mice treated with or without AF. As shown in Figure 1, the AUC _insulin _during the 90 min study period in high-fat fed mice was decreased compared with mice fed normal diet (128 ± 26 vs. 275 ± 41 nmol/l). However, AF treatment had no effect on impaired glucose tolerance induced by high-fat feeding (Figure 2a and 2c). AF did not affect and/or increase glucose tolerance in mice fed normal diet (Figure 2b and 2d).

### Islet studies

**Figure 3:**
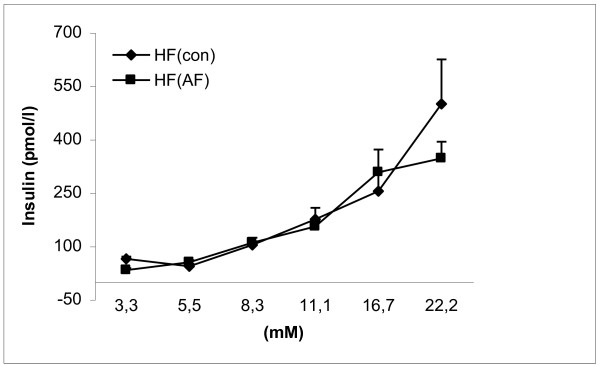
**Insulin secretion in isolated islets from AF treated C57BL/6J for 8 weeks**. Glucose-stimulated insulin secretion was determined during a 60-min incubation of islets isolated from C57BL/6J mice after 8 weeks of a high-fat diet. Results are expressed as means ± SE for each group and glucose concentration (n = 4).

Insulin secretion over a wide range of glucose concentrations was similar in isolated islets from control and AF-treated high-fat-fed mice (Figure 3). Similar results were also observed in isolated islets from normal diet-fed mice after 8 weeks of treatment with AF (data not shown).

## Discussion

Our previous results demonstrated that AF increased glucose tolerance and insulin secretion in NMRI mice after being administered through a gastric gavage in a single high dose of 75 or 150 mg per mouse. Furthermore, in these mice, AF enhanced the increase in plasma levels of the gut hormone, GLP-1. In the present study, we examined whether long-term administration of AF at a more moderately high dose influences glucose-stimulated insulin secretion in C57BL/6J mice. C57BL/6J mice have been shown to be susceptible to developing glucose intolerance more readily than other strains when maintained on a high-fat diet [14-16] and were the selected model in the current study.

It was observed that AF did not influence glucose tolerance and insulin secretion in C57BL/6J mice fed a high-fat diet (Figure 1, 2, 3). In this study, AF was administered to the mice, fed either a high-fat or normal diet for 8 weeks, via drinking water. The dose of AF was 1.5 g/kg/day, or around 30 mg/mouse/day. This dose is much lower than the one used in our earlier single and high dose study [13]. It would be possible that the current dose of 30 mg/mouse/day was not sufficient to change glucose tolerance and insulin secretion. However, high dose of AF, which might have an effect, may not have practical application either as a drug or a health food additive. Beside the different mouse models and the high dose used [13], the routes of administration of AF were also different in these two studies. In the previous study [13], AF was given through a gastric gavage tube placed in the stomach of the mice, while AF was administered through drinking water in the present study. It is not sure if these might have influenced the effective concentration of AF in the circulating blood, which may be important for the biological activity of AF. On the other hand, if the major *in vivo *physiological function of AF is by its antioxidant activity [12], it might not improve glucose tolerance under these conditions since it has been reported that high fat does not cause oxidative stress [17]. It is also known that fatty acids acutely amplify glucose-induced insulin secretion from the pancreatic β-cell, but they become harmful when present at elevated levels for prolonged periods of time due to their deleterious effects on β-cell function including inhibition of insulin secretion, the so-called lipotoxicity [17]. That is, the possible positive effect of AF on glucose tolerance might have been blunted due to high-fat diet feeding. A high-fat diet has been shown to induce hyperglycemia, hyperinsulinemia, hyperlipidemia, obesity and glucose intolerance in C57BL/6J mice (Table 1) [15,16,18]. Thus, an additional drug might be needed together with AF in order to overcome the strong changes in pathophysiology in C57BL/6J mice challenged with a high-fat diet compared to acute administration of a single high dose of AF in normal NMRI mice [13]. It should be noted also that though AF is energy-negative feeding mice at 1.5 g/kg/day apparently did not affect the weight gain (Table 1).

AF has recently been reported to have several health beneficial effects including anti-inflammatory and anti-cancer effects [19-21] beside its antioxidant effect [12]. Though no indication of improved glucose tolerance was evident, this study further confirmed our early observations [22,23] that AF is a safe sugar to consume as no adverse effect was observed in the current study.

## Conclusions

With C57BL/6J mice as model which is liable to develop glucose intolerance when fed on a high-fat diet, 1,5-Anhydro-D-fructose (AF) at 1.5 g/kg/day, administered through drinking water for 8-weeks, did not affect body weight gain or food and water intake fed on either high-fat or normal diet. No difference in basal blood glucose or insulin levels between AF-treated and control group was found. These indicate that AF as a food antioxidant does not apparently interfere with sugar metabolism in mice.

## Competing interests

The authors declare that they have no competing interests.

## Authors' contributions

JM was the major person performed the experiments using the 1,5-anhydro-D-fructose prepared and provided by SY. The experiments were designed and discussed by JM, SY and BA. All these work were performed at Department of Medicine, Lund University, Lund, Sweden.

## Pre-publication history

The pre-publication history for this paper can be accessed here:

http://www.biomedcentral.com/1472-6823/10/17/prepub
